# Phytochemical analysis and evaluation of the cytotoxic, antimicrobial and antioxidant activities of essential oils from three *Plectranthus* species grown in Saudi Arabia

**DOI:** 10.1186/s12906-018-2302-x

**Published:** 2018-08-10

**Authors:** Ramzi A. Mothana, Jamal M. Khaled, Omar M. Noman, Ashok Kumar, Mohamed F. Alajmi, Adnan J. Al-Rehaily, Mine Kurkcuoglu

**Affiliations:** 1 Department of Pharmacognosy, College of Pharmacy, King Saud University, P.O. Box 2457, Riyadh, 11451 Saudi Arabia; 2 Departments of Botany and Microbiology, College of Science, King Saud University, Riyadh, 11451 Saudi Arabia; 3 Vitiligo Research Chair, College of Medicine, King Saud University, Riyadh, Saudi Arabia; 4 Department of Pharmacognosy, Faculty of Pharmacy, Anadolu University, Eskisehir, Turkey

**Keywords:** *Plectranthus* species, GC, GC/MS, Essential oil, Anticancer, Antimicrobial, Antioxidant

## Abstract

**Background:**

Cancers and microbial infections are still a major health problem, therefore research on new anticancer and antimicrobial agents ought to be continued. Natural products including essential oils from medicinal plants continue to be an important resource to manage various diseases. Thus, the particular objectives of this study are to investigate the chemical composition, cytotoxic, antimicrobial and antioxidant activities of three *Plectranthus* species namely *P. cylindraceus* Hocst. ex Benth., *P. asirensis* JRI Wood and *P. barbatus* Andrews grown in Saudi Arabia*.*

**Methods:**

The essential oils of the three *Plectranthus* species were obtained by hydrodistllation and analyzed using GC/FID and GC-MS. The essential oils were further assessed for their cytotoxic, antimicrobial and antioxidant activities. Determination of the cytotoxic activity was carried out against Hela, HepG2 and HT-29 cancer cell lines by utilizing MTT-assay. The antimicrobial activity was assessed against six bacterial and fungal strains by using broth micro-dilution assay. In addition, the antioxidant activity was evaluated utilizing the DPPH and β-Carotene-linoleic acid assays.

**Results:**

The GC/FID and GC-MS analysis led to the identification of 59, 60 and 42 compounds representing 89.0% 95.0 and 97.1% of the total essential oils of *P. cylindraceus, P. asirensis* and *P. barbatus*, respectively. The essential oils were characterized by a high content of oxygenated sesquiterpenes in *P. cylindraceus*, sesquiterpene hydrocarbons in *P. asirensis* and monoterpene hydrocarbons in *P. barbatus* where maaliol (42.8%), β-caryophyllene (13.3%) and α-pinene, (46.2%) were the predominant compounds. Additionally, the oils particularly of *P. cylindraceus* and *P. barbatus* exhibited remarkable cytotoxic and antimicrobial activities with IC_50_-values between 3.8 and 7.5 μg/mL and MIC-values ranging from 0.137 to 4.40 mg/mL. Moreover, the oils showed moderate to high radical scavenging and antioxidative activities ranging from 52 to 75% at the highest concentration of 1 mg/mL.

**Conclusions:**

The observed results back the suggestion that these three *Plectranthus* species represent a promising source of cytotoxic and antimicrobial agents.

## Background

Malignant diseases as well as infections caused by microorganisms and parasites are still a serious menace to public health, in spite of the great development in human medicine [1, 2]. Consequently, research on new anticancer and antimicrobial agents ought to be continued. Natural products including essential oils from medicinal plants continue to be a substantial resource to treat different diseases, particularly in developing countries [3–6]. Recently, WHO (World Health Organization) reported that 80% of people worldwide rely on phytomedicine for some aspect of their primary health care needs [5]. As indicated by WHO, around 21,000 plant species have the potential for being utilized as medicinal plants [5]. Approximately 3000 volatile oils are reported in the literature however; only 10% of those are used in different pharmaceutical, food and cosmeceutical industries [7]. Comprehensive endeavors have been carried out in assessing the cytotoxic and antimicrobial capacity of essential oils [3, 4, 7, 8]. Thus, this study is a part of our ongoing investigations on plants containing essential oils growing in the Arabian Peninsula.

The genus *Plectranthus* (Family: Lamiaceae) speaks to a great and widely distributed collection of species with a variety of folkloric uses. This genus involves a group of about 300 species, spread in tropical and suptropical territories of Asia, Australia and Africa [9, 10]. The genus *Plectranthus* is represented in Saudi Arabia by seven species distributed in the South of the Kingdom [9]. The species of this genus are well-known medicinal species used extensively for the treatment of different illnesses. A considerable assortment of traditional therapeutic uses of the genus *Plectranthus* in Central and East Africa, India China and Brazil have been reported. The majority of uses are for intestinal disorders and liver stress, respiratory disturbances, heart diseases, malaria and central nervous system disorders [11–15]. *Plectranthus* species are rich in diterpenoids as well as essential oils which are reported to be responsible for various pharmacological activities such as antibacterial, antifungal, cytotoxic and antiplasmodial activities [10, 12, 13, 16–21]. The current investigation is an aspect of our ongoing works on volatile oils and their pharmacological activities of Saudi medicinal herbs. Thus, the particular aims of this study are to provide detailed data on the chemical composition, cytotoxic, antimicrobial and antioxidant activities of three *Plectranthus* species namely *P. cylindraceus* Hocst. ex Benth. (Synonym: *P. montanus* Benth.)*, P. asirensis* J.R.I. Wood (Synonym: Coleus arabicus Benth.) and *P. barbatus* Andrews (Synonym: *P. barbatus* var. *barbatus*) grown in Saudi Arabia*.*

## Methods

### Plant materials

Aerial parts of the three *Plectranthus* species were collected from Al-Baha region, Saudi Arabia in December 2016 and authenticated at the Pharmacognosy Department, College of Pharmacy, King Saud University (KSU). Voucher samples (KSU 16263, 15,779 and 15,732) were deposited for the three species *P. cylindraceus* Hocst. ex Benth.*, P. asirensis* JRI Wood and *P. barbatus* Andrews respectively at the Pharmacognosy Department, College of Pharmacy, KSU.

### Extraction of volatile constituents of *Plectranthus* species

The volatile oils were extracted once from 500 g of the dried and ground, leaves and branches of each *Plectranthus* species by water-distillation (3 h), utilizing a Clevenger-type apparatus. Finally, the obtained oils were desiccated utilizing anhydrous Na_2_SO_4_ and kept at low temperatures (+ 4 °C) for further experiments.

### GC/MS analysis

Gas chromatographic analysis was performed on a 5975 Gas Chromatograph coupled with-mass spectrometer (Agilent, USA; SEM Ltd., Istanbul, Turkey). Innowax FSC column (60 m × 0.25 mm, 0.25 μm film thickness) was utilized as stationary phase while helium was utilized as a moble phase (0.8 mL/min). The volume injected was 0.1 μL with a split ratio of 40:1. The oven temperature of the GC was intialy set at 60 °C for 10 min, then increased to 220 °C at a rate of 4 °C/min, held constant for 10 min and thereafter increased to 240 °C at a rate of 1 °C/min. The temperatures of the injector and transfer line were set at 250 and 280 °C respectevely. MS detection was performed at 70 eV with scan mass range m/z 35–450.

### GC/FID analysis

Analyses were carried out on an Agilent Technologies 6890 N GC system with flame ionization detector. The temperature of the FID was programmed to 300 °C. The same column utilized in GC–MS experiments as well as the same operational conditions were performed to a triplicate. Simultaneous auto injection was done to obtain equivalent retention times. The quantification (relative percentages) of the identified compounds was calculated from the FID peak area percent normalization.

### Identification of compounds

The volatile oil components were identified by comparing the mass spectra with those of similar compounds in Adams library [22], Mass Finder terpenoid library [23], Wiley GC/MS Library [24] and our own Baser Library of Volatile Oil Constituents, on the basis of their retention indices. The identification was completed by comparing the retention times with authentic reference standards and by comparing the retention index (RRI) relative to C_8_-C_30_ of *n*-alkanes under the same above mentioned operating conditions [25]. The results are given as mean percentage ± standard deviation (SD) (*n* = 3) as shown in Table 1.

**Table 1:** Chemical composition of the essential oils of *Plectranthus cylindraceus* (A), *P. asirensis* (B) and *P. barbatus* (C)

No.	Compounds	RRI	A %	B %	C %	Identification
1	**α-Pinene**	1032	0.2	**8.6**	**46.2**	t_R_, MS
2	α -Thujene	1035	tr	5.9	–	MS
3	α-Fenchene	1072	–	–	tr	t_R_, MS
4	Camphene	1076	0.2	0.1	4.5	t_R_, MS
5	β-Pinene	1118	0.1	1.9	3.3	t_R_, MS
6	Sabinene	1132	0.1	1.5	–	t_R_, MS
7	Thuja-2,4 (10)-diene	1138	–	tr	0.2	MS
8	δ-3-Carene	1159	tr	–	–	t_R_, MS
9	Myrcene	1174	0.1	1.3	0.5	t_R_, MS
10	α-Terpinene	1188	2.3	0.4	–	t_R_, MS
11	Limonene	1203	0.2	0.2	1.8	t_R_, MS
12	1,8-Cineole	1213	–	–	2.7	t_R_, MS
14	β-Phellandrene	1214	0.4	tr	–	t_R_, MS
15	*p*-mentha-1, 3, 6-triene	1215	–	tr	–	MS
16	*cis*-Anhydrolinalool oxide	1220	tr	–	–	MS
17	(Z)-3-Hexenal	1225	–	tr	–	t_R_, MS
18	(Z)-β-Ocimene	1246	–	tr	–	t_R_, MS
19	γ-Terpinene	1255	0.3	1.0	0.1	t_R_, MS
20	(E)-β-Ocimene	1266	–	0.8	–	t_R_, MS
21	*p*-Cymene	1280	5.5	0.4	1.1	t_R_, MS
22	Terpinolene	1290	tr	0.5	0.1	t_R_, MS
23	(*Z*)-3-Hexenol	1391	–	0.3	–	t_R_, MS
24	3-Octanol	1393	0.1	tr	–	MS
25	α-Thujone	1438	–	tr	–	MS
26	*p*-Cymenene	1452	0.1	tr	0.1	MS
29	β-Thujone	1457	–	0.1	–	MS
30	1-Octen-3-ol	1459	0.2	0.5	0.1	t_R_, MS
31	*trans*-Sabinene hydrate	1474	0.1	–	–	t_R_, MS
32	*cis*-Linalool oxide (*Fur.*)	1479	tr	–	–	MS
33	Bicycloelemene	1495	–	tr	–	MS
34	α-Copaene	1497	0.1	0.2	0.7	MS
35	**Camphor**	1532	**7.2**	–	0.3	t_R_, MS
36	β-Bourbonene	1535	tr	0.3	tr	t_R_, MS
37	α-Gurjunene	1544	0.1	–	–	MS
38	*cis*-α-Bergamotene	1550	tr	–	–	MS
39	Linalool	1553	2.3	1.3	0.5	t_R_, MS
40	*cis*-Sabinene hydrate	1556	0.1	–	–	t_R_, MS
41	α-Bergamotene	1577	0.2	0.3	–	MS
42	Pinocarvone	1586	tr	–	0.1	MS
44	Aristolene	1589	tr	–	–	t_R_, MS
45	Bornyl acetate	1590	–	0.2	2.3	t_R_, MS
46	β-Elemene	1600	0.1	tr	–	MS
47	Calarene	1610	1.2	–	–	t_R_, MS
48	Terpinen-4-ol	1611	0.8	3.3	1.4	t_R_, MS
49	**β-Caryophyllene**	1612	1.3	**13.3**	0.5	t_R_, MS
50	Aromadendrene	1628	–	0.4	–	MS
51	Selina-5,11-diene	1634	tr	–	–	MS
52	*trans-p*-Menth-2,8-dien-1-ol	1638	0.1	–	–	MS
53	Myrtenal	1648	–	1.2	0.2	MS
55	Sesquisabinene	1660	0.6	0.3	–	t_R_, MS
56	Alloaromadendrene	1661	0.1	4.4	–	MS
57	*trans*-Pinocarveol	1664	–	0.8	0.9	t_R_, MS
58	*cis*-Verbenol	1667	–	–	0.1	Ms
59	(Z)-**β**-Farnesene	1668	–	tr	–	MS
60	α-Humulene	1687	0.1	1.0	0.2	t_R_, MS
61	*trans*-Verbenol	1690	–	–	0.6	MS
62	δ-Selinene	1700	0.1	–	–	MS
63	α-Terpineol	1706	0.1	0.8	2.0	t_R_, MS
64	**Borneol**	1719	0.1	2.1	**20.7**	t_R_, MS
65	Germacrene D	1726	0.5	2.0	–	MS
66	β-Selinene	1742	1.0	–	–	MS
67	α-Selinene	1744	tr	–	–	MS
68	Sesquicineole	1747	5.3	–	–	MS
69	*p*-Mentha-1,5-dien-8-ol	1747	–	0.5	0.6	MS
70	**Bicyclogermacrene**	1751	0.8	**7.4**	–	MS
71	δ-Cadinene	1772	–	0.5	–	t_R_, MS
72	γ-Cadinene	1776	0.2			MS
73	(E)- α-Bisabolene	1786	0.1	–	–	MS
74	Myrtenol	1796	–	2.6	1.1	MS
75	α-Cadinene	1811	–	–	0.2	MS
76	*cis*-Calamenene	1853	0.5	–	–	
77	*p*-Cymen-8-ol	1864	–	–	0.2	t_R_, MS
78	Ascaridole	1889	0.4	–	–	MS
79	*trans*-Sesquisabinene hydrate	2000	0.2	5.2	–	MS
80	Isocaryophyllene oxide	2001	–	0.2	–	MS
81	Caryophyllene oxide	2008	0.8	4.7	0.8	t_R_, MS
82	**Maaliol**	2012	**42.8**	0.2	–	MS
83	(*E*)-Nerolidol	2041	–	0.8	–	t_R_, MS
84	Humulene epoxide-II	2071	–	0.4	0.7	MS
85	1, 10-diepi- Cubenol	2080	0.4	–	–	MS
86	Globulol	2096	1.1	0.5	–	MS
87	*cis*-Sesquisabinene hydrate	2097	–	0.8	–	MS
88	Cumin alcohol	2113	0.1	–	–	t_R_, MS
89	Rosifoliol	2122	0.2	–	–	MS
90	**Spathulenol**	2144	3.3	**8.7**	–	t_R_, MS
91	Cedrol	2148	–	–	4.0	MS
92	β-Bisabolol	2170	–	2.0	–	MS
93	T-Cadinol	2191	1.3	–	0.3	MS
94	Valerenal	2194	3.2	–	–	MS
95	α-Bisabolol	2232	1.6	–	–	MS
96	*trans*-α-Bergamotol	2241	–	1.5	–	MS
97	β-Eudesmol	2255	–	2.2	0.6	MS
98	Intermedeol	2260	–	–	tr	MS
99	Selina-11-en-4α-ol	2273	–	–	tr	MS
100	Caryophylladienol I	2316	0.1	0.1	–	MS
101	Caryophylladienol II	2324	0.6	0.3	tr	MS
102	Caryophyllenol I	2353	–	–	tr	MS
103	Caryophyllenol II	2392	–	0.4	tr	MS
104	Phytol	2622	–	0.2	tr	MS
105	Heptacosane	2700	–	0.1	–	MS
106	Hexadecanoic acid	2931	–	tr	–	MS
	Monoterpene hydrocarbons		**9.7**	**22.1**	**57.8**	
	Oxygenated monoterpenes		**11.1**	**10.1**	**31.2**	
	Sesquiterpene hydrocarbons		**7.1**	**36.1**	**1.6**	
	Oxygenated sesquiterpenes		**60.8**	**25.6**	**6.4**	
	Other compounds		**0.3**	**1.1**	**0.1**	
	**Total**		**89.0**	**95.0**	**97.1**	

Boldface means major compounds (compounds with high content or high persentage)

*RRI* Relative retention indices calculated against n-alkanes. %; calculated from the FID chromatograms, *tr* Trace (< 0.1%); Identification method: t_R_, identification based on the retention times (t_R_) of genuine compounds on the HP Innowax column; MS, identified on the basis of computer matching of the mass spectra with those of the Wiley and MassFinder libraries and comparison with literature data

### Determination of anticancer activity on human cancer cell lines

#### Cancer cell lines and culture

The assay was carried out on three tumor cell lines, human cervical cancer (HeLa), human hepatocellular liver carcinoma (HepG2) and human colon cancer (HT-29) which were obtained from ATCC (USA). HeLa, HepG2 and HT-29 cells were maintained in DMEM/high glucose supplemented with 2 mM l-glutamine, 10% fetal calf serum and 1% penicillin-streptomycin.

#### In vitro cytotoxic activity by MTT test

The cytotoxic activity of the essential oils was assessed on cell viability using MTT-assay. This test measures the cellular viability based on reduction capacity of the viable cells to convert 3-[4,5-dimethylthiazol-2-yl]-2,5-diphenyltetrazolium bromide (MTT) to formazan crystals as previously described [26, 27] with some modifications. Briefly, cells were seeded (2 × 10^4^ cells/well) in growth medium (DMEM) into flat-bottom microdilution plates of 96 wells (in quintuplicates) and incubated at 37 °C in a 5% CO_2_ incubator for 24 h. The essential oils were added at different concentrations to each well while the medium in control wells was replaced by SFM (serum free medium) containing an equivalent volume of dimethyl sulfoxide (DMSO) and incubated for further 24 h. The concentrations tested ranged from 1.97 to 250 μg/mL. After that SFM was removed and 100 μL of MTT (0.5 mg/ml) was added to each well and incubated at 37 °C for another 3 h to estimate cell viability. MTT solution was removed and 100 μL isopropanol was added to each well to dissolve the formed purple formazan crystals with shaking for 1 h at room temperature. At the end, the plates were read at 549 nm using a microplate reader (ELX 800; Bio-Tek Instruments, Winooski, VT, USA). The induced cytotoxicity was calculated by comparing the optical density (OD) values against those in control wells. Cytotoxicity was expressed as IC_50_-value which was calculated as the concentration of the volatile oil inhibiting cell viability by 50%. Dasatinib was utilized as a positive control. All measurements were performed in triplicate and the means and standard errors were calculated.

### Determination of antimicrobial activity

#### Test microorganisms

The bacterial and fungal microorganisms used in this study were the Gram-positive bacteria *Bacillus subtilis* (ATCC 6633), *Streptococcus mutans* (ATCC 25175), *Brevibacillus laterosporus* wild strain, the Gram-negative bacteria *Salmonella typhi* wild strain, and the fungal strains *Candida albicans* (ATCC 60193) and *Cryptococcus neoformans* wild strain.

#### Minimal inhibitory concentrations (MIC)

The MIC values of the three essential oils against three Gram-positive, one Gram-negative and two fungi strains were estimated using micro-well dilution method as described previously [28] with modifications. With sterile round-bottom 96-well plates, duplicate two-fold serial dilutions of each essential oil (100 μL/well) were prepared in the suitable broth (Mueller Hinton or Sabouraud Dextrose broth) containing 5% (*v*/v) DMSO to establish a range of concentrations (20 to 0.156 μL/mL) of essential oil. 100 μL (1 × 10^6^ CFU/mL) of the bacterial or fungal suspension which was previously prepared in the proper broth, was then added in each well except those in column 10, 11 and 12, which used as negative controls for oil, saline and media sterility. The last well in each plate was served for bacterial or fungal growth without essential oil. After that the 96-well plates were incubated at the appropriate temperature for each strain for 24 h. The MIC of each oil was specified as the lowest essential oil concentration exhibiting no detectable bacterial or fungal growth. Gentamycin and nystatin with a range of concentrations of 125 to 0.97 μg/mL were used as a positive controls. For the determination of MBC and MFC (minimal bactericidal concentration and minimal fungicidal concentration), part of the liquid (5 μL) from each well that exhibited no growth was taken and incubated on agar plates at 37 °C for further 24 h. The lowest concentration that revealed no visible bacterial or fungal growth was considered as MBC or MFC.

### Determination of antioxidant activity

#### DPPH radical-scavenging activity

The antioxidant activity of the essential oils was measured using 2,2-diphenyl-1-picrylhydrazyl (DPPH) as described previously by Brand-Williams et al., 1995 [29]. This assay assess the radical scavenging activity of the DPPH by the tested sample. Five concentrations (10, 50, 100, 500 and 1000 μg/mL) of each essential oil were prepared. Then 500 μL of the essential oil was added to 125 μL DPPH methanol solution (1 mM) and 375 μL methanol and incubated for 30 min at room temperature in the dark. At the, the anti- DPPH radical-scavenging activity was measured by recording the absorbance at λ = 517 nm and calculated using the following formula:

$$ \%\mathrm{of}\ \mathrm{anti}-\mathrm{radical}\ \mathrm{activity}=\mathrm{Abscontrol}-\mathrm{Abssample}/\mathrm{Abscontrol}\ \mathrm{X}\ 100 $$

#### β-Carotene bleaching test

The antioxidative activity of the three *Plectranthus* essential oils was investigated by using the β-carotene bleaching test as described by Mothana et al., 2012 [30] with modification. β-carotene solution (1000 μL) which was prepared by dissolving 200 μg in 1 mL chloroform, was added to a flask containing a solution of 200 μL of Tween-20 and 20 μL of linoleic acid. Using rotatory evaporator the chloroform was removed, 100 mL of distilled water was added and the mixture was vigorously shaken for 2 min. 200 μL of the volatile oil (1000 μg/mL) was added to 2 mL of the β-carotene-linoleic acid emulsion and incubated at 40 °C for 2 h. Finally the absorbance was measured at 470 nm at 30 min intervals, by a UV-spectrophotometer (UV mini-1240, Shimadzu, Japan). As a positive control, rutin at a concentration of 1000 μg/mL was utilized. The antioxidative activity was estimated by using the following formula:

1 $$ \mathrm{Antioxidant}\ \mathrm{activity}\ \left(\%\right)=\left(\mathrm{Abs}0-\mathrm{Abst}\right)/\left(\ {\mathrm{Abs}}^{{}^{\circ}}0-{\mathrm{Abs}}^{{}^{\circ}}\mathrm{t}\right)\ \mathrm{X}\ 100 $$

where, Abs0 and Abs°0 are the absorbencies measured at zero time of incubation for the essential oil and blank samples, respectively. Abst and Abs°t are the absorbencies for essential oil and blank samples, respectively, at 120 min.

#### Statistical analysis

Results are demonstrated as means ± standard deviations (SD) for experiments carried out in triplicate. The data were analyzed by one-way ANOVA using Tukey test (IBM, SPSS, statistics 25). Significance difference was designated by probability values of *P* ≤ 0.05.

## Results

The obtained volatile oils were colorless and aromatic. The three *Plectranthus* species yielded 0.18%, 0.05 and 0.10% (*w*/w) of the oils respectively.

### GC/FID and GC/MS analysis

**Fig. 1:**
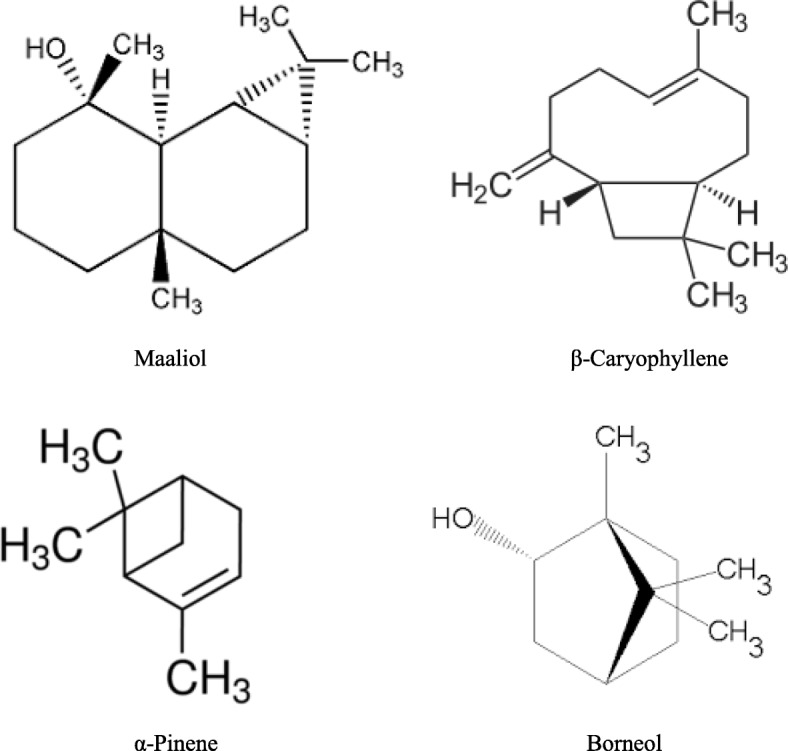
Chemical structures of the main constituents identified in the essential oils of the three investigated *Plectranthus* species

The results of the GC/FID and GC/MS are demonstrated in Table 1. It showed the chemical composition of the analyzed oils, retention indices, percentages and identification methods. The identified compounds are listed in order of their elution on the HP Innowax column. The GC/FID and GC-MS investigation drove to the identification of 59, 60 and 42 compounds representing 89.0, 95.0 and 97.1% of the total essential oil of *P. cylindraceus, P. asirensis* and *P. barbatus* respectively. Maaliol (42.8%) was the major constituent in the volatile oil of *P. cylindraceus* while β–caryophyllene (13.3%) was the predominant constituents followed by α-pinene and spathulenol (8.6 and 8.7%) in *P. asirensis* essential oil (Table 1, Fig. 1). The *P. barbatus* essential oil showed the predominance of α-pinene (46.2%) followed by borneol (20.7%) as major components (Table 1, Fig. 1).

### Cytotoxic activity

As shown in in Table 2, the essential oils of the *Plectranthus* species demonstrated a noteworthy cytotoxic activity against all cancer cell lines with IC_50_ values ranging between 3.88 to 7.51 μg/mL. The most promising cytotoxic results against HeLa, HepG2 and HT-29 cancer cell lines were observed with the essential oil of *P. cylindraceus* with IC_50_ values of 3.97, 3.88 and 3.91 μg/mL, respectively which were stronger than the positive control dasatamib (Table 2).

**Table 2:** Cytotoxic activity of the essential oils of *Plectranthus* species (μg/mL)

Test samples	IC_50_ values (μg/mL)		
	HeLa	HepG2	HT-29
*P. cylindraceus*	3.97 ± 0.13 d	3.88 ± 0.06 c	3.91 ± 0.15 c
*P. asirensis*	7.51 ± 0.48 b	7.19 ± 0.74 a	6.82 ± 0.65 b
*P. barbatus*	4.97 ± 0.15 c	4.99 ± 0.17 b	4.93 ± 0.1 c
Dasatamib	5.57 ± 0.17 a	4.05 ± 0.21 b	5.24 ± 0.18 b

*HeLa* human cervical cancer, *HepG2* human hepatocellular liver carcinoma, *HT-29* human colon cancer. In the columns of IC_50_, means ± SD with different letters notification are significant at (*P* < 0.05) (*n* = 3)

### Antimicrobial activity

MICs, MBCs and MFCs of the essential oils are shown in Table 3. As demonstrated in Table 3, the essential oils exhibited variable degrees of bacterial and fungal growth inhibition (MIC-values: 0.137–4.40 mg/mL). The most sensitive microorganisms were the bacteria strain *Brevibacillus laterosporus* and the fungal strain *Cryptococcus neoformans*. The essential oils of *P. cylindraceus* and *P. barbatus* demonstrated the strongest antimicrobial activity with MIC values ranging between 0.137 and 0.55 mg/mL against almost microorganisms. MBC or MFC values were also exhibited and obtained one time higher than that of MIC’s with all essential oils (Table 3).

**Table 3:** Minimal inhibitory concentrations (MIC), minimal bactericidal concentration (MBC) and minimal fungicidal concentration (MFC) of the essential oils of *Plectranthus* species (mg/mL)

Microorganisms		Activity	*P. cylindraceus*	*P. asirensis*	*P. barbatus*	RA^a^	RA^b^
Bacteria	*B. subtilis*	MIC	0.275	0.275	0.275	7.8	NT
		MBC	0.55	0.55	0.55	15.6	NT
	*S. mutans*	MIC	1.10	2.20	1.10	7.8	NT
		MBC	2.20	4.40	2.20	15.6	NT
	*B. laterosporus*	MIC	0.137	0.275	0.137	3.9	NT
		MBC	0.275	0.55	0.275	7.8	NT
	*S. typhi*	MIC	0.275	0.55	0.137	3.9	NT
		MBC	0.55	1.10	0.275	7.8	NT
Fungi	*C. albicans*	MIC	0.55	2.20	0.55	NT	3.5
		MFC	1.10	4.40	1.10	NT	7.0
	*C. neoformans*	MIC	0.275	0.275	0.275	NT	3.5
		MFC	0.55	0.55	0.55	NT	7.0

*B. subtilis*: *Bacillus subtilis* ATCC 6633, *S. mutans*: *Streptococcus mutans* ATCC 25175, *B. laterosporus*: *Brevibacillus laterosporus* wild strain, *Salmonella typhi* wild strain, *C. albicans*: *Candida albicans* ATCC 60193 and *C. neoformans*: *Cryptococcus neoformans* wild strain, *RA*^a^, Reference antibiotic (Gentamycin), *RA*^b^ Reference antibiotic (Nystatin). Values are given as mg/ml for essential oils and μg/ml for standard antibiotics, *NT* not tested

#### Antioxidant activity

The results of the radical scavenging and antioxidative activities are given in Table 4. In the β-carotene-bleaching model system, the three *Plectranthus* essential oils showed variable powers to inhibit the β-carotene bleaching at a concentration of 1000 μg/mL with total antioxidative values of 71, 52 and 62% for *P. cylindraceus*, *P. asirensis* and *P. barbatus* respectively (Table 4). In addition, the results of the DPPH radical scavenging method exhibited a strong free radical scavenging activity for the essential oil of *P. cylindraceus* at the highest concentration 1000 μg/mL (75%) followed by *P. barbatus* and *P. asirensis* which showed 68 and 55% respectively (Table 4).

**Table 4:** Antioxidant activity and free radical scavenging activity of the essential oils of *Plectranthus* species

Samples	^a^Total antioxidant Activity in % (1000 μg/mL)	Free Radical Scavenging Activity in % (DPPH-radical scavenging assay)				
		10	50	100 (μg/mL)	500	1000
*P. cylindraceus*	71.1 ± 4.5a	25.1 ± 4.2c	39.4 ± 3.5a	50.2 ± 4.3	70.5 ± 3.9a	75.9 ± 4.0b
*P. asirensis*	52.5 ± 4.9a	15.6 ± 3.9b	20.4 ± 3.8c	36.9 ± 1.6a	46.5 ± 4.1	55.1 ± 2.9b
*P. barbatus*	62.9 ± 5.1b	21.1 ± 4.8b	30.5 ± 4.1a	44.5 ± 3.1b	60.6 ± 3.8c	68.2 ± 3.2a
Ascorbic acid	NT	81.4 ± 5.2	87.2 ± 3.8	91.0 ± 4.5c	93.0 ± 3.8a	94.1 ± 4.4c
Rutin	88.2 ± 5.1b	NT	NT	NT	NT	NT

^a^β-carotene bleaching assay, *NT* not tested. In the columns, means ± SD with different letters notification are significant at (*P* < 0.05) (*n* = 3)

## Discussion

Research on anticancer and antimicrobial agents from natural sources should be continued in order to discover novel, more effective and less expensive drugs. Consequently, in our continuing search for valuable and promising natural products from Saudi medicinal plants, this study was carried. In the current study, we analyzed the chemical composition of the essential oils of three *Plectranthus* species namely *P. cylindraceus, P. asirensis* and *P. barbatus* by using GC/FID and GC/MS. The current study was further extended to investigate the anticancer, antimicrobial and antioxidant activities of these essential oils.

As far as we know, this study represents the first investigation on the cytotoxic, antimicrobial and antioxidant activities of these essential oil of *P. asirensis*. The existing knowledge about the chemical composition, cytotoxic, antimicrobial and antioxidative activities of volatile oils of *Plectranthus* species grown in Saudi Arabia is in many cases limited.

In *P. cylindraceus*, the oxygenated sesquiterpenes (60.8%) were determined as the major group, among which maaliol (42.8%) was found to be the main constituent (Table 1). Oxygenated monoterpenes (11.1%) followed in this essential oils as a second major group with camphor (7.2%) as a main component. Our results are partly in agreement with a recent published report by Khan and co-workers on the essential oil of *P. cylindraceus* grown in Saudi Arabia who revealed the predominance of oxygenated sesquiterpenes too but with patchouli alcohol (55.5%) as a major component. Our results were not in agreement with previous studies on the essential oil of *P. cylindraceus* grown in Yemen, Oman or Ethiopia where thymol (68.5%), carvacrol (46·8%) and camphor (40.9%) were identified as the major constituents respectively [31–33]. Moreover, our results of the essential oil of *P. asirensis* was not in agreement with the data of a recent published study which revealed that *P. asirensis* oil was mainly characterized by the presence of monoterpenoids (90.7%) where thymol (66.0%), followed by γ-terpinene (14.0%) represented the major constituents [34]. Our results revealed that chemical composition of the essential oil of *P. barbatus* was dominated by the presence of monoterpene hydrocarbons (57.8%) followed by oxygenated monterpenes (31.2%). α-pinene (46.2%) as well as borneol (20.7%) dominated in this oil and demonstrated the representatives for both monoterpenoid groups. To the best of our knowledge this is the first report on the chemical composition of essential oil of *P. barbatus* grown in Saudi Arabia. Our results are partly in agreement with a previous reports on *P. barbatus* essential oil which showed α-pinene (22.2 and 19.3%) as a major component [35, 36].

Almost these variations in the chemical composition of the volatile oils of the *Plectranthus* species are attributed to differences in the geographical environmental factors, e.g. climate, soil and altitude as well as the phenological stage, time of collection, and extraction techniques of the plant species and this certainly contributed to produce a spectacular chemical composition of the oils.

In the current study, we observed promising cytotoxic, antimicrobial and antioxidant activities for the three essential oils. The cytotoxic activity of the oils was determined against three types of cancers (Hela, HepG2 and HT-29) using MTT test. All essential oils particularly those of *P. cylindraceus* and *P. barbatus* showed remarkable cytotoxic activity against all tested three cancer cell lines. In general, literature data on *Plectranthus* essential oils cytotoxicity are still scarce. Contrary to our results, a work presented by Ali et al. [31] highlighted a weak cytotoxic activity of *P. cylindraceus* essential oil (18% at 100 μg/mL) against HT-29 tumor cells. Furthermore, in a recent study done by Amina and co-workers [10], maaliol was isolated from the ethanolic extract of *P. cylindraceus* and showed the highest cytotoxic activity among the isolated compounds against MBDMB321, HT1080 and Hela cancer cell lines with IC_50_ values of 28.1, 25.9 and 27.1 μg/mL respectively. Thus, the great cytotoxic activity of the *P. cylindraceus* essential oil could be mainly attributed to maaliol which predominated with 42.8%.

In addition, α-pinene, borneol and β-caryophyllene have been reported to show in vitro cytotoxicity to different cancer cells e.g. HepG2, MCF-7, MDA-MB-468 and UACC-257 cancer cell lines. Consequently, the cytotoxic activity of the oils of *P. asirensis* and *P. barbatus* could probably be attributed to these constituents which were characterized as dominant constituents [37–41].

Little has been reported on the antimicrobial activity of *P. asirensis* and *P. barbatus* essential oils however, some papers highlighted the antimicrobial activity of *P. cylindraceus* essential oil. Our results showed that among the tested microbial strains, *B. laterosporus* and *C. neoformans* were found to be the most sensitive to the *Plectranthus* essential oils. Works by Marwah and co-workers [32] and Asres and co-workers [33] on the essential oil of *P. cylindraceus* grown in Oman or Ethiopia exhibited similar or higher antimicrobial activity with MIC-values in the range of 7–62 μg/mL against various bacterial and fungal strains. Some previous studies support the hypothesis that certain essential oil components e.g. α-pinene, borneol, 1,8-cineole, maaliol and prostantherol produced from various plant species have antimicrobial activity [42–45].

## Conclusion

Our results revealed that the chemical composition of the essential oils of the three *Plectranthus* species varied from each another. The GC and GC/MS analysis identified a high content of maaliol (42.8%) in *P. cylindraceus*, β-caryophyllene (13.3%) in *P. asirensis* and α-pinene (46.2%) in *P. barbatus*. Furthermore, the results clearly provide evidence that the three essential oils possess remarkable cytotoxic and antimicrobial but moderate antioxidant activities. These data back the suggestion that the investigated *Plectranthus* species represent a hopeful source of cytotoxic and antimicrobial compounds. Further experiments are required to separate the active principles as well as in vivo investigations to prove their efficacy and safety for clinical use.

## Abbreviations

ATCC: American type culture collection
CFU: Colony-forming units
GC: (gas chromatography)
GC-FID: (gas chromatography-flame ionization detector)
GC-MS: (gas chromatography-coupled mass spectrometry)
MBC: Minimum bactericidal concentration
MFC: Minimum fungicidal concentration
MIC: Minimum inhibitory concentration
MTT: [3-(4.5-dimethylthiazolyl)-2.5-diphenyl-tetrazolium bromide
RI: retention index
